# Symptomatic Epilepsy*Read as part of Symposium on Epilepsy at Buffalo Academy of Medicine, January 14, 1913.

**Published:** 1913-05

**Authors:** Edward Affleck Sharp

**Affiliations:** Buffalo, N. Y.; Neurologist, Craig Colony of Epileptics


### Symptomatic Epilepsy*

BY EDWARD AFFLECK SHARP, M.D.
Buffalo, N. Y.
Neurologist, Craig Colony of Epileptics

THE term Symptomatic Epilepsy is used in the present instance to emphasize the fact that all forms of epilepsy have their cause, and that some definite lesion, either of a gross or histological nature, is present in the brain or its membranes in practically every case.

The conception of the organic basis of the disease dates from the time of Galen, who thought that epilepsy was due to a peculiar alteration of the brain and of the nerves coming from it.

The term idiopathic or genuine epilepsy, while still used by many writers, is usually defended on the ground that its etiology is unknown or indefinite. Oppenheim still retains the term "genuine epilepsy," but does not place much importance in such a designation, altho he thinks an organic basis is not to be assumed for all cases, and that in neuropathic and psychopathic individuals a strong psychic shock may result in the development of epilepsy. Attacks of this character would probably be more correctly classified under the term psycho-genetic convulsions.

Numerous instances are on record where lesions of considerable extent, in the central nervous system, have been found at autopsy in cases which clinically were considered as idiopathic epilepsy and which apparently gave no history nor showed physical signs pointing to such a lesion.

While the term "idiopathic" is still used to describe certain form of epilepsy it is quite certain that some organic alteration is present as the cause of the epilepsy and that the lesions can be demonstrated in many cases.

An enumeration of the lesions found in epileptics would include almost every variety of pathological change which occur in the brain or its membranes. Many of these are of a histological nature, such as the sub-pial gliosis of Chaslin or Alzheimer, which has been found in about 40 per cent. of the cases of so-called genuine epilepsy, and the diffuse sclerosis of the cornua Ammonis found in about 50 per cent. of such cases, or the changes are of a cellular nature less easily demonstrable. In other cases gross lesions of considerable extent are found, such as thickened meninges, oedema of the pia, areas of sclerosis, porencephaly, cysts, new growths, hemorrhages, atrophy of lobes or convolutions, dilitation of the ventricles, etc.

---

*Read as part of Symposium on Epilepsy at Buffalo Academy of Medicine, January 14, 1913.

It is these gross lesions which will form the principal part of the present discussion and which will be supplemented by a lantern-slide demonstration by Dr. Munson, showing some of the more prominent lesions which have been found in the Craig Colony cases.

Most of the lesions found in epileptics have originated in early life or before birth as a result of encephalitis, meningitis, trauma, hemorrhages, toxaemias, hereditary syphilis or developmental defects from hereditary or other influences, and the epilepsy is to be considered as a symptom-complex or syndrome occurring as a result of these lesions.

The acute infectious diseases of childhood may be accompanied by a mild meningo-encephalitis, and this undoubtedly has some influence in producing some of the fine histological changes in the brain. Alcoholism and syphilis in the parents are important factors in producing certain changes which result in a susceptibility to convulsive disorders.

In studying the family histories of a large number of epileptics one of the most striking features is the frequency with which some hereditary factor exists, and this appears equally prominent in many of the cases which have some of the gross lesions mentioned above. Epilepsy does not belong to the direct hereditary diseases, nor does it act as a Mendelian unit in its method of transmission, but a predisposition is inherited producing an unstable nervous system and then other factors act as exciting causes. Of interest in this connection is the recent report by Marchand and Petit of a case of epilepsy occurring five days after birth in a child born of an imbecile and epileptic mother. The child died eight days after birth and autopsy showed diffuse lesions of meningo-encephalitis. Clinically the infant showed no signs of cerebral disease prior to the development of the epilepsy.

Epilepsy may develop after middle life as a result of the infantile encephalopathies, but it is more frequently the result of alcoholism, syphilis or other toxaemias, or from new growths, abscess, traumatic lesions, hemorrhage, sclerosis, etc. The senile cases usually develop on the basis of an arterio-sclerosis or diffuse cortical changes and meningeal adhesions.

Several factors are necessary for the production of an epileptic seizure. The organic lesions of the brain are constantly present and yet the attacks may occur at long intervals. In other cases equally extensive lesions are found in cases which never become epileptic. Some other factor is therefore necessary, acting as an exciting cause, to set off the discharge in the susceptible cortex.

The definite periodicity of the attacks, with an interparoxysmal period of relative freedom from symptoms, would suggest some disturbance of metabolism or some toxic agent developing in the body in excess of the ability of the organism to eliminate or neutralize the poison, and which acts, either directly on the cortical cells or through the vascular supply, as an exciting cause for the attack.

This disturbance may originate in the gastro-intestinal tract or in some of the ductless glands or other organs, and toxins are generated which have a particularly irritating effect on a cerebral cortex already lowered in its power of resistance by the presence of some organic lesion, or on an unstable nervous system the product of a neuropathic ancestry.

In children the vaso-motor system is usually very impressionable and the slightest lesion in the brain may result in a hyperaemic or anaemic condition which acts as an exciting cause for an attack.

The influence of the pituitary body in the pathogenesis of epilepsy has been pointed out by Cushing in his recent monograph. His conclusions are that undue excitability of the cerebral cortex may be a consequence of posterior lobe insufficiency, and that local scars on the cortex may prevent access of the cerebro-spinal fluid with its hypophyseal secretion which is essential to functional stability.

It is doubtful if these toxins would be able to produce convulsions or psychic disturbances except in the presence of some such lesions in the brain. Even the so-called para-syphilitic epilepsy of Fournier, which was considered a pure dynamic epilepsy from the toxins of syphilis, is probably always associated with fine histological changes.

It is on this basis that epilepsy is considered as practically always symptomatic of some organic lesion or cellular change in the cerebral cortex. If the term "idiopathic" is to be used it should be limited to the cases which develop on an hereditary basis and in which only cellular changes occur which are beyond the power of the microscope to demonstrate, but which nevertheless are assumed to be present.

Some of the cases with the most extensive lesions have shown very few or no clinical symptoms during the interparoxysmal period, and the attacks have been similar to the so-called idiopathic cases.

If one has the opportunity of studying the case during an attack, some evidence of the organic nature of the epilepsy may frequently be seen.

In an institution like the Craig Colony, where a large number of seizures are observed, one is impressed with the great variety of the clinical manifestations of the convulsions in the different patients and with the constancy with which the attacks in any individual case remain more or less of a fixed type and to such an extent that the patient is easily identified by the character of his seizure.

A definite order of invasion of the seizure, the convulsive movement starting in one extremity and always spreading to other parts in the same regular manner, or remaining unilateral or localized to one extremity as in the Jacksonian type of seizure, usually indicates a focal lesion, or one of greater severity, over the cortical representation of the initial movement. In some cases a definite aura will indicate the site of the initial discharge, and the presence of an olfactory, auditory, gustatory or visual aura may serve as a valuable localizing sign.

Temporary exhaustion paralysis is usually found in the extremity or part most involved in the convulsion and this may be the only evidence that a localized or unilateral organic lesion is present.

Usually the lesions above mentioned show a special predilection to affect the motor cortex of one or both sides or the deeper structures in the path of the motor fibres, producing the various forms of infantile cerebral palsy. Over 10 per cent. of the cases at the Craig Colony are of this type.

When a hemiplegia is manifest and is accompanied by contractures, spasticity, deformities, etc., there is no difficulty in recognizing the organic nature of the affection. In some of the cases of infantile cerebral palsy there has been almost complete recovery from the hemiplegia, and the epilepsy is the only outward manifestation of the former injury to the brain. Careful examination, however, will usually reveal some evidence of the former hemiplegia. The exhaustion paralysis following the attack has already been mentioned. During the interparoxysmal period there may be a slight rigidity of the extremity which is only found by careful testing and comparison with the healthy member, or there is a slight weakness or early fatigue of the part on exertion.

Athetoid and associated movements may be present or may be brought out only after exercise and fatigue, and a slight awkwardness of the hand on the side formerly hemiplegic may be seen when the patient is asked to pick up small objects after exercising the extremity.

The tendon reflexes are usually exaggerated on the side formerly hemiplegic, and the abdominal reflexes may be absent or more easily exhausted on the hemiplegic side.

The Babinski toe sign and other signs of pyramidal tract involvement may be present during the interparoxysmal period and they may become temporarily increased immediately after an attack.

The character of the attack may also give some evidence of the location of the lesion, and the experimental and clinical studies of Ziehen, Rothmann and others have shown that when the attack consists of a tonic and a clonic stage, the tonic component of the attack is of sub-cortical origin while the clonic component is of cortical origin.

From experimental evidence it is found that an irritation of the motor cortex without involving the sub-cortical structures is accompanied by a clonic convulsion without the preceding tonic stage, and theoretically a case having a convulsion of this character would be a favorable one for surgical intervention.

A particular clinical variety of the clonic convulsion from cortical irritation is known as "Epilepsia partialis continua" or the Syndrome of Kojevnikoff.

Lesions in the cerebellum sometimes produce tonic spasms of the extremities, and in pure "cerebellar fits" the attacks are always tonic. If clonic convulsions of the cerebral type occur in cerebellar lesions they are due to the increased intracranial pressure acting on the motor cortex.

The fact that extensive lesions are frequently found at autopsy in the brains of epileptics and that these lesions have been sometimes overlooked, shows the necessity and importance of making a thorough examination for the clinical signs pointing to such lesions, and not to consider epilepsy as a simple neurosis amenable to bromide or other sedative treatment.

Many of these lesions, altho easily accessible surgically, show the futility of attempts to operate on them, as the surface lesion is frequently only a small portion of the actual damage, and in many cases the lesions are multiple and deeply seated. But, if these organic lesions in the brain cannot be removed, they can be made less active as factors in the production of epileptic seizures by reducing to a minimum the exciting causes of toxic nature which set off the discharge. It is for this reason that so much importance is placed on dietetic and hygienic measures in the treatment of epilepsy.

The influence of these extra-cerebral factors, as exciting causes for the convulsions, is shown in the occasional brilliant results obtained by removing sources of reflex irritation or by correcting disturbed functions of the gastro-intestinal tract, the ductless glands or other organs.

REFERENCES.

Neurologisches Centralblatt 1912. Nos 11 and 20.
Revue Neurologique 1911/i p. 368.
Revue Neurologique 1912/ii p. 110.
Gaz. des Hosp. 1912, No. 91.
Cushing. Pituitary Body.
Clark. Boston Med. and Surg. Jour., 1912.

---

Dr. M. Milton Portiss, of Chicago, read a paper on "Hyperchlorhydria" before Elkhart Co. Med. Soc., February 6, 1913. (Jour. Ind. State Med. Ass'n, Mch.) Meat diet contra-indicated; vegetable less irritating. Based on experiments on dogs, Dr. Portiss has prepared following table:

| | Time in Stomach Hr. Min. | Am't of Secretion, c.c. |
|---|---|---|
| 250 c.c. potato | 1:30 | 12 |
| 250 c.c. spinach | 1:40 | 15 |
| 250 c.c. carrots | 1:25 | 14 |
| 250 c.c. milk | 2:10 | 15 |
| 1 lb. meat (finely divided) | 4:15 | 32 |
| 1 lb. meat (in chunks) | 7:30 | 63 |
| 1 lb. meat (in chunks) plus Hyoscin-Hydrobromid | 6:30 | 52 |
| 1 lb. meat (in chunks) plus Morphin gr. ½ | 7:00 | 56 |
| 1 lb. meat (in chunks) plus Atropin gr. 1/50 | 4:20 | 25 |
| 1 lb. meat (in chunks) plus Strontium Bromid, 15 g. | 4:15 | 27 |
| 1 lb. meat (in chunks) plus Strontium Bromid, 30 gr. | 3:30 | 17 |

---

SEA SICKNESS. E. Scheppelmann has prepared an interesting monograph on this subject published by Walter Rothschild, Berlin. *American Medicine* comments: "It has been observed that a person immersed in a salt water bath on a badly rolling ship, loses all sense of the ship's motion." We venture to disagree, though our evidence was lost along with the rest of the contents of the tub. We had no contents left.

